# From Palpitations to Prevention: Timely Recognition of Biventricular ACM Preventing Sudden Cardiac Death

**DOI:** 10.1155/cric/3342504

**Published:** 2026-03-04

**Authors:** Jasraj Singh, Fadi W. Adel, Horng H. Chen

**Affiliations:** ^1^ Department of Cardiovascular Medicine, Mayo Clinic, Rochester, Minnesota, USA, mayo.edu

**Keywords:** arrhythmia, arrhythmogenic cardiomyopathy, arrhythmogenic right ventricular cardiomyopathy, sudden cardiac death, ventricular tachycardia

## Abstract

Sudden cardiac death (SCD) is a prevalent and significant health concern which may be preceded by palpitations and presyncope in a young patient. Of the arrhythmogenic causes of SCD, arrhythmogenic cardiomyopathy (ACM) is rare but important with a high morbidity and mortality. Here, we present a classic case of biventricular (BiV) ACM in a man in his 30s who presented with progressive palpitations and syncope found to have nonsustained ventricular tachycardia and suspicious cardiac imaging findings. Diagnosis was confirmed according to the 2024 European Task Force Criteria: T wave inversions in V1–V3 without a right bundle branch block (major criterion); regional right ventricular (RV) systolic dyskinesis with both reduced RV systolic function by CMR (RVEF 34% and normal 42%–66%); and enlarged RV by indexed RV EDV by CMR (131 mL/m^2^) (major criterion). LV global longitudinal strain was reduced on transthoracic echocardiography at −17% (normal more negative than −18%) (minor criterion) and T wave inversions in left precordial leads (V4–V6) (in the absence of complete LBBB) (minor criterion). With timely intervention, including ICD placement and sotalol initiation, the patient made a good recovery. This case serves as a critical reminder that recognizing the subtle yet telling signs of BiV‐ACM can mean the difference between life and sudden cardiac death.

## 1. Introduction

Sudden cardiac death (SCD) is a significant public health concern, leading to 450,000 deaths annually. The most common causes of SCD are coronary artery disease (70%) and cardiomyopathy (15%), with inherited arrhythmias contributing to 2% of cases annually [1]. Of the inherited arrhythmias, arrhythmogenic cardiomyopathies (ACMs), including arrhythmogenic right ventricular cardiomyopathy (ARVC) and its biventricular form, are distinct though rare entities which must be considered when evaluating the young patient with palpitations and presyncope given the high potential for progression to SCD. In this report, we describe the case of a man in his 30s who presented with progressively frequent palpitations and presyncope, who was diagnosed and treated for a new diagnosis of biventricular arrhythmogenic cardiomyopathy (BiV‐ACM).

## 2. Case Presentation

A man in his late 30s presented to the clinic for evaluation of progressive presyncopal dizziness and chest tightness which were intermittently occurring for the preceding 7 years. He endorsed associated palpitations, especially with preceding caffeine intake. Though he had not lost consciousness with any presyncopal episodes, he had fallen once. He had no headache, shortness of breath, abdominal pain, nausea, vomiting, paresthesia, or weakness. He had no family history of sudden death or early onset heart disease. He did not drink alcohol to excess and was a nonsmoker with no illicit drug use history. Physical examination was significant for mild pectus excavatum, mild arthrogryposis and dystrophic nails, and on cardiac auscultation regular rate and rhythm with occasional extrasystoles apparent on auscultation and palpation of the radial pulse.

Prior to referral, he was hospitalized for observation after an episode of presyncope. He was found to have elevated cardiac troponins which did not continue to rise after initial measurement, and a significant finding of precordial T wave inversions from V2 to V6 with occasional PVCs on ECG. His inpatient cardiac monitor reportedly recorded frequent ventricular ectopy of uncertain morphology. Upon discharge, a 30‐day cardiac event monitor demonstrated a baseline rhythm of normal sinus rhythm without any premature ventricular contractions (PVCs) or premature atrial contractions (PACs). However, he did have an emergent finding of one symptomatic run of nonsustained ventricular tachycardia (NSVT) at 200 bpm for nine beats.

Repeat electrocardiogram in our clinic demonstrated normal sinus rhythm with sinus arrhythmia, normal axis, and T wave inversions in V1–V5 and normal intervals including QRS duration (Figure 1a). Chest radiograph demonstrated no cardiomegaly (Figure 1b).

Figure 1(a) Electrocardiogram demonstrating precordial T wave inversions in the absence of right bundle branch block. There were no Epsilon waves. (b) Chest X‐ray demonstrating normal cardiac silhouette and no evidence of pulmonary vascular congestion.

**Figure:**
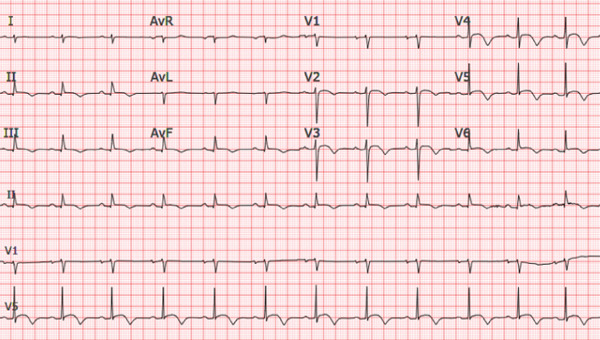
(a)

**Figure:**
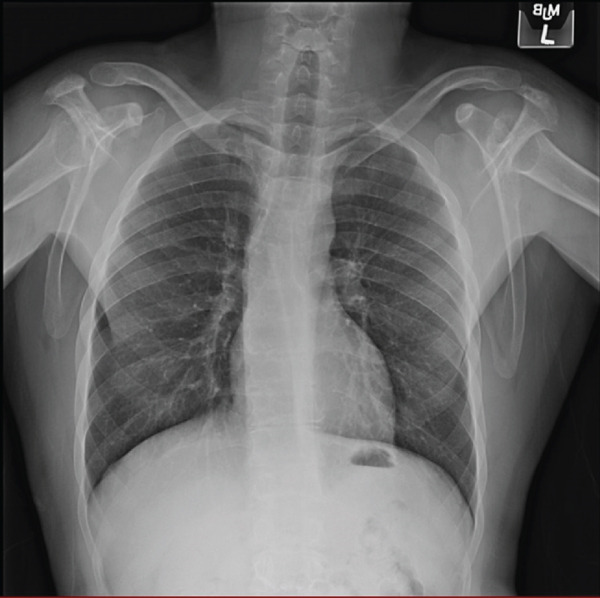
(b)

We obtained a transthoracic echocardiogram which revealed a mildly enlarged RV chamber size based on ventricular measurements (base and mid RV diameters were 45 and 40 mm, respectively, and base‐apex length of 84 mm) and reduced RV systolic function based on a reduced RV fractional area change of 22.76% (Figure 2). Tissue Doppler imaging revealed tricuspid lateral annulus systolic velocity of 0.08 m/s (below the normal threshold of 0.1 m/s), tricuspid annulus systolic excursion (TAPSE) by M‐Mode of 17 mm, and a tricuspid regurgitant velocity of 2.1 m/s. Echocardiogram also revealed decreased longitudinal peak systolic RV strain (−16%), normal chamber pressures, and no evidence of significant valvulopathy. While he had a preserved left ventricular ejection fraction of 54%, he had mild left ventricular apical hypokinesis, and LV global longitudinal strain was reduced on transthoracic echocardiography at −17% (normal more negative than −18%).

Figure 2Echocardiogram demonstrating findings in (a) systole and (b) diastole demonstrating right ventricular dilatation and reduced systolic function (calculated fractional area change of 22.76%).

**Figure:**
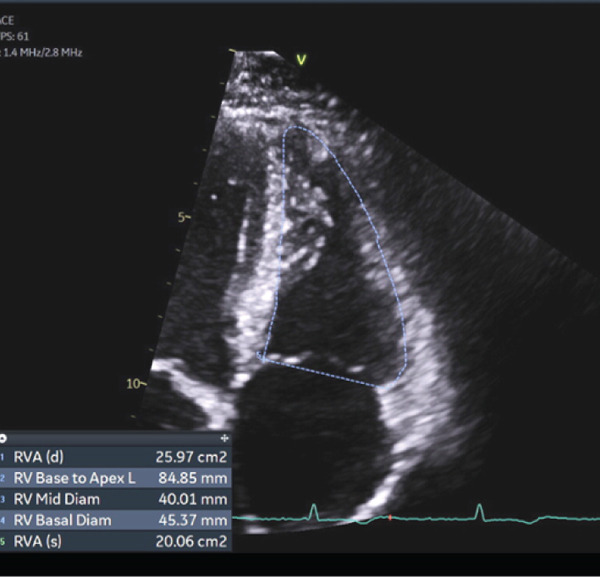
(a)

**Figure:**
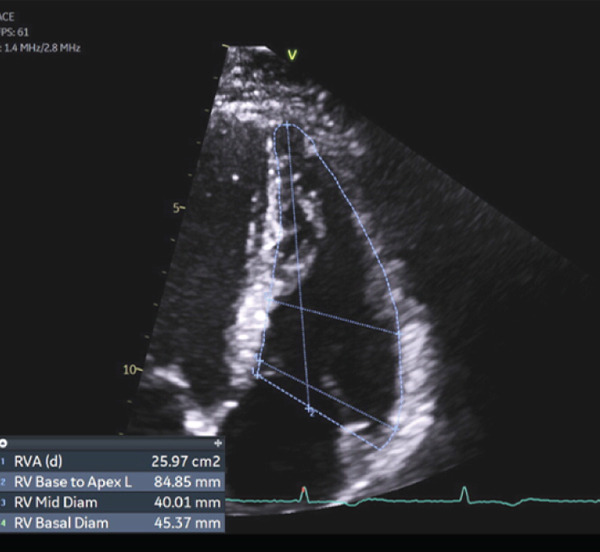
(b)

Due to high suspicion for ACM, cardiac MRI was pursued, revealing regional dyskinetic systolic function of the RV free wall and LGE along the free mid‐apical RV wall (Figure 3). Additionally, cardiac MRI demonstrated RV dilatation by elevated diastolic index of 131 mL/m^2^ and reduced right ventricular ejection fraction of 34%. Further, the patient had left ventricular apical thinning and hypokinesis, along with some evidence of delayed enhancement in the anterior apex, possibly correlating with the ECG showing T wave inversions in the precordial leads. These findings confirmed the suspected diagnosis of BiV‐ACM, for which the patient was emergently admitted for expedited management (Table 1).

Figure 3Cardiac magnetic resonance imaging demonstrating late gadolinium enhancement of the (a) mid‐apical right ventricular free wall and (b) regional systolic dyskinesis of the same region. The arrow designates the region of dyskinesis.

**Figure:**
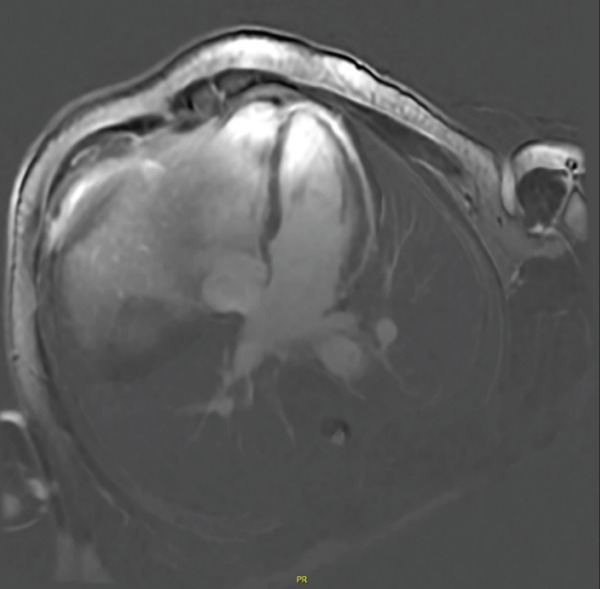
(a)

**Figure:**
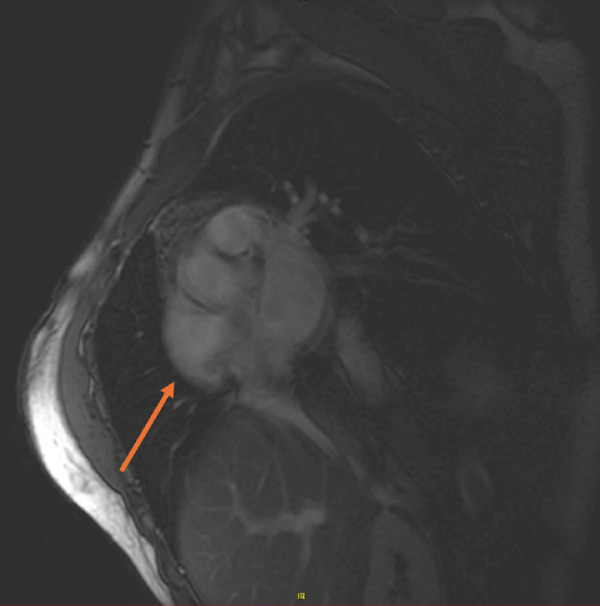
(b)

**Table 1:** Diagnostic criteria for BiV‐ACM and our patient′s findings.

Criterion (Padua 2020/TF‐2024)	Definition/threshold	Patient finding (exact)	Meets criterion?
CMR—RV morpho‐functional (major)	Indexed RVEDV and/or RVEF beyond major cutoffs (major if RVEDVi ≥ 110 mL/m^2^ and/or RVEF ≤ 40*%* per TF/Padua imaging thresholds).	RVEDVi 131 mL/m^2^; RVEF 34%.	Yes—major
Echocardiography—RV regional wall‐motion + FAC (major)	Regional RV akinesia/dyskinesia/aneurysm plus reduced FAC (major if FAC ≤ 33*%* with regional abnormality).	Regional RV dyskinesis; RV‐FAC 22.8%.	Yes—major
ECG—repolarization (major)	T wave inversion in right precordial leads (V1–V3 or beyond) in the absence of complete RBBB = major.	Precordial T wave inversion extending to V1–V4/V1–V5 (reported V2–V6 in places); no complete RBBB documented.	Yes—major
Ventricular arrhythmia—documented VT/NSVT (major if typical morphology)	VT/NSVT with LBBB‐type morphology and specified axis on 12‐lead or frequent PVC burden (major if morphology consistent with RV origin and/or high burden).	Documented NSVT (9 beats ≈200 bpm) on event monitor; 12‐lead morphology not explicitly documented in current manuscript.	Partially—documented but not scored as major without 12‐lead morphology
CMR—tissue characterization (LGE) (supportive/structural weight)	Nonischemic LGE in RV free wall or characteristic “ring‐like” LV scar supports diagnosis (Padua/TF emphasize tissue characterization).	Subepicardial/mid‐apical RV free‐wall LGE; some LV delayed enhancement/apical hypokinesis.	Yes—supportive/increases confidence
Left‐ventricular phenotype (Padua biventricular/ALVC elements)	LV regional dysfunction/LGE consistent with biventricular phenotype increases diagnostic scoring and changes risk/management framing.	LV apical hypokinesis; LV GLS −17%; LV delayed enhancement reported.	Yes—present (biventricular phenotype)
Genetics—pathogenic variant (major)	Presence of pathogenic/likely pathogenic ACM gene variant (desmosomal etc.) = major. VUS does not qualify.	DSG2 variant—classified as VUS.	No

While inpatient, genetic testing was undertaken and it was significant for a heterozygous variant of unknown significance in desmoglein‐2 (DSG2). A shared decision‐making discussion was held regarding rhythm management, and based on his definitive diagnosis of BiV‐ACM by Padua and ETF 2024 guidelines, his documented NSVT history, and significant observed PVC burden, we estimated his 5‐year risk as 45% using the Cadrin–Tourigny model [2]. Due to his high risk of ventricular arrhythmias, we recommended placement of a dual‐chamber implantable cardioverter‐defibrillator (ICD) for primary prevention [3]. He underwent placement of a dual‐chamber ICD for primary prevention of SCD and initiation of sotalol for ventricular ectopy.

He recovered well and was discharged on sotalol after observation. On cardiology follow‐up at 12 months, he has continued to feel well and has had no complications related to his medical therapy, and serial device interrogations have shown no subsequent episodes or shocks. Subsequent echocardiography has demonstrated an LVEF of 55%–56% without regional wall abnormalities.

## 3. Discussion

ACM is often an inherited cardiomyopathy characterized by the progressive replacement of ventricular myocardium with fibrofatty tissue. In most cases, inherited genetic mutations affecting desmosomes and adherens junctions compromise intramyocardial cell–cell adhesion, predisposing to myocardial detachment, cell death, and subsequent fibrosis. This pathological process leads to myocardial atrophy and predisposes patients to ventricular arrhythmias and SCD [4–6]. The most commonly affected region is the “triangle of dysplasia” between the anterior pulmonary infundibulum, right ventricular apex, and right ventricular infero‐posterior wall, though expansion beyond this region is often seen. Left ventricular involvement becomes increasingly more likely in advanced stages [5, 7].

Occurring at a rate of one in 1000–5000 persons, ACM (previously ARVC) and its biventricular form, BiV‐ACM, are rare arrhythmogenic causes of SCD. Affected patients often initially present with palpitations or presyncope—as in our case—or sudden death at around 25 years of age [5]. Sixteen culprit genetic mutations have been linked to ARVC, with most being autosomal dominant mutations affecting desmosomal proteins such as plakophilin 2 (PKP2) and DSG2 [7]. However, no genetic substrate is found in up to 40% of cases [4, 7].

The clinical course of ACM is conceptualized in three phases, with the first “concealed” phase characterized by subtle right ventricular structural changes. As fibrofatty tissue expands throughout the myocardium, patients enter the “electrical phase” where electrocardiographic findings—most commonly precordial T wave inversions, terminal QRS prolongation, premature ventricular complexes, and ventricular tachycardia—begin to manifest. Finally, the “structural” phase occurs when substantial structural changes lead to isolated right or biventricular heart failure [7, 8].

Diagnosis is challenging and multifaceted based upon the 2020 Padua Guidelines and most recently the 2024 European Task Force Guidelines which incorporate family history, electrocardiographic abnormalities, imaging findings, pathologic features (if biopsy or autopsy findings are available), and genetic information [7, 9–11]. Given the low diagnostic yield of myocardial biopsy, a combination of electrical and imaging findings is often used in concert with family history to make the diagnosis as was the case with our patient. In addition to the electrical abnormalities listed above, QRS fragmentation (characterized by small right precordial deflections at the start of the QRS complex superimposed on the R wave) and epsilon waves are also seen on electrocardiogram. In some cases, as in our patient′s, concomitant left ventricular involvement can be identified by T wave inversion in the precordial and inferior leads and low voltage in peripheral leads. Laboratory testing in many cases will often yield elevated troponins [12]. Echocardiography showing right ventricular motion abnormalities is an essential pillar of diagnosis and can be detected by cardiac MRI. Due to the spatial limitations of MRI technology, direct visualization of fibrofatty replacement is not required, and LGE has been found to be nonspecific [7, 13].

SCD prevention with placement of ICDs is the cornerstone of ARVC management for high‐risk patients such as those presenting with ventricular tachycardia or sudden death [5, 6]. Risk stratification of patients presenting with palpitations, ectopy, or presyncope can be challenging, with some studies suggesting younger age, inducibility of arrhythmia at electrophysiology study, and high premature ventricular complex burden are predictors of future sudden death. Pharmacological treatment options, such as beta‐blockers, amiodarone, and sotalol, are used to manage arrhythmias, although their efficacy varies [14–16]. In our patient′s case, sotalol was effective at controlling ectopic ventricular ectopy. Emerging therapies, including radiofrequency ablation and potential future options like stereotactic radiotherapy, are being explored [6].

In summary, BiV‐ACM is a rare cause of arrhythmogenic SCD which affects young patients and may present as presyncope or palpitations as shown by our patient′s case. Maintaining a high clinical suspicion and pursuing a thorough cardiac structural, electrical, and functional evaluation is essential to making the diagnosis. Pharmacologic management is often necessary, and ICD placement is essential in high‐risk patients.

## Author Contributions

J.S.: conceptualization, data collection, writing—original draft, and writing—review and editing. F.W.A.: conceptualization, investigation, data collection, and writing—review and editing. H.H.C.: conceptualization, writing—review and editing, and supervision.

## Funding

The authors received no specific funding for this work.

## Consent

Written patient consent was obtained to use his chart data for research and study reporting as part of the Minnesota Research Authorization program.

## Conflicts of Interest

The authors declare no conflicts of interest.

## Data Availability

Data sharing is not applicable to this article as no datasets were generated or analyzed during the current study.
